# Transbronchial lung biopsy versus transbronchial lung cryobiopsy in critically ill patients with undiagnosed acute hypoxemic respiratory failure: a comparative study

**DOI:** 10.1186/s12890-022-01966-4

**Published:** 2022-05-04

**Authors:** Shiyao Wang, Yingying Feng, Yi Zhang, Ye Tian, Sichao Gu, Xiaojing Wu, Yiming Feng, Ling Zhao, Min Liu, Dan Wang, Ying Li, Zheng Tian, Shumeng Wang, Xu Huang, Guowu Zhou, Qingyuan Zhan

**Affiliations:** 1 Department of Pulmonary and Critical Care Medicine, Center of Respiratory Medicine, China-Japan Friendship Hospital; National Center for Respiratory Medicine; National Clinical Research Center for Respiratory Diseases; Institute of Respiratory Medicine, Chinese Academy of Medical Sciences, 2 Yinghuayuan East Street, Chaoyang District, Beijing, 100029 China; 2 Department of Pathology, China-Japan Friendship Hospital, Beijing, 100029 China; 3 Department of Radiology, China-Japan Friendship Hospital, Beijing, 100029 China

**Keywords:** Cryobiopsy, Acute respiratory failure, Diagnostic yields, Complications

## Abstract

**Background:**

In patients with acute hypoxemic respiratory failure whose diagnosis is not established after initial evaluation, obtaining a histopathological diagnosis may improve the patients’ prognosis. This study aims to compare the safety profile and diagnostic yields between transbronchial lung biopsy (TBLB) and transbronchial lung cryobiopsy (TBLC) in these patients.

**Methods:**

A retrospective comparative study was conducted in a 26-bed intensive care unit over a 5-year period. The consecutive patients with acute hypoxemic respiratory failure who underwent TBLB or TBLC were included to determine the potential etiology. Patients characteristics, procedure related complications, pathological and multidisciplinary discussion (MDD) diagnostic yields, treatment modification and 28-day survival were analyzed. Prognostic factors were identified by Cox regression analysis.

**Results:**

Forty-five and 25 consecutive patients underwent TBLB and TBLC, respectively. The patients underwent TBLC were more critical. There was no significant difference in overall procedure related complications of patients underwent TBLB and TBLC [15.6% (7/45) vs 28.0% (7/25), *p* = 0.212]. The rate of pathological diagnostic yield [72.0% (18/25) vs 37.8% (17/45), *p* = 0.006], MDD diagnostic yield [84.0% (21/25) vs 55.6% (25/45), *p* = 0.016] and subsequent treatment modification [84.0% (21/25) vs 57.8% (26/45), *p* = 0.025] in patients underwent TBLC were significantly higher than those in patients underwent TBLB. Multivariate analysis revealed that MDD diagnosis [HR 0.193 (95% CI 0.047–0.792), *p* = 0.022] and treatment modification [HR 0.204 (95% CI 0.065–0.638), *p* = 0.006] may be prognostic protective factors.

**Conclusions:**

TBLC can lead to an increased chance of establishing a diagnosis, which could significantly improve the patients’ prognosis, with an acceptable safety profile.

## Introduction

Acute hypoxemic respiratory failure is a general reason for patients' admission to the intensive care unit (ICU). For patients who meet the definition of acute respiratory distress syndrome (ARDS), the morbidity and mortality rate is as high as 35 to 60% [1, 2]. The etiology of acute hypoxemic respiratory failure is diverse, with lung infection being the most common etiology. However, there are many other etiologies such as interstitial lung disease, organizing pneumonia, cancer, and drug induced lung injury. Many of the etiologies are treatable, the prognosis of this group of patients can be improved if the diagnosis is established by biopsies [3]. Therefore, biopsy should be performed to obtain pathology for definitive etiologies for acute hypoxemic respiratory failure patients with uncertain diagnosis.

Obtaining pathology in patients with acute hypoxemic respiratory failure, especially in mechanically ventilated patients, is a challenging clinical problem. Several studies have shown that critically ill patients with undiagnosed pulmonary infiltrations may benefit from surgical lung biopsy (SLB) [3–6]. However, the complication rate of SLB in these patients is more than 20%, and it may be as high as 59% in mechanically ventilated patients. The main complications are persistent air leak and bleeding [7]. The tissue specimens obtained from transbronchial lung biopsy (TBLB) are small, with limited pathological diagnostic value. And the risk of postprocedure pneumothorax up to 20% [7]. In summary, the uses of both SLB and TBLB in critically ill patients have limitations.

Transbronchial lung cryobiopsy (TBLC) is a new method of biopsy that has been widely used for the diagnosis of interstitial lung disease (ILD). 50–87% of ILD patients can be diagnosed by TBLC, and the diagnostic yield can reach 90% when combined with multidisciplinary discussions (MDD). The incidence of pneumothorax is about 10%, and the incidence of moderate to severe bleeding is about 4–28% in ILD patients [8]. Our team has attempted to perform TBLC to obtain pathology in patients with non-resolving acute respiratory distress syndrome (ARDS). The complications were acceptable, and most patients obtained pathological diagnosis [9]. Therefore, we retrospectively analyzed the safety and diagnostic yield of TBLB and TBLC in critically ill patients with undiagnosed acute hypoxemic respiratory failure, aiming to reveal the value of TBLC in this group of patients.

## Method

### Patients

**Fig. 1:**
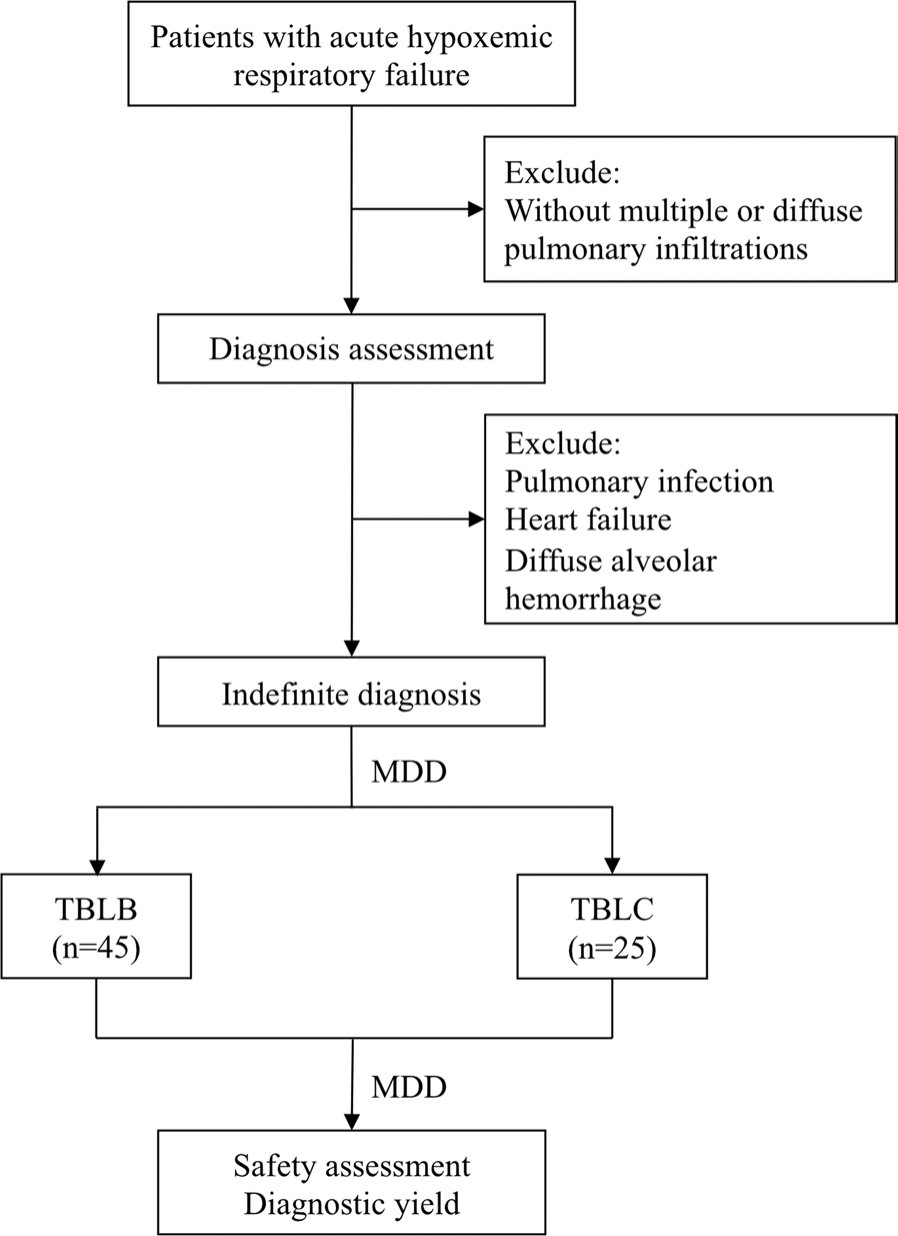
Flow diagram of patient selection and comparative content in the study. MDD: multidisciplinary discussions; TBLB: transbronchial lung biopsy; TBLC transbronchial lung cryobiopsy

We reviewed the medical records of all patients hospitalized in the 26-bed tertiary medical intensive care unit (MICU) between September 1, 2016, and September 30, 2021. The process and conditions of patient selection are shown in Fig. 1. A total of 45 consecutive patients underwent transbronchial lung biopsy (TBLB), and 25 consecutive patients underwent transbronchial lung cryobiopsy (TBLC). All patients were admitted for acute hypoxemic respiratory failure. The initial assessment failed to determine the etiology of pulmonary infiltration, such as pulmonary infection, heart failure, or diffuse alveolar hemorrhage by laboratory tests and bronchoalveolar lavage fluid (BALF) analysis. The decision of procedure was made by multidisciplinary discussion (MDD) according to the patients’ clinical conditions, imaging performance, and safety considerations. Written informed consent was obtained from all patients prior to the procedure.

### Procedure

All 45 patients receiving TBLB were operated bedside in the MICU with bronchoscopy inserted via nasal route under NIPPV support or endotracheal tube (ETT) under IPPV support. The ventilator would be set as FiO_2_ to 1.0 and EPAP/PEEP to 0 cm H_2_O. The target segment for the biopsy was decided by MDD prior to the procedure. The biopsy position was determined by radial probe endobronchial ultrasound (RP-EBUS) (EU-ME1, Olympus, Tokyo, Japan) or 1–2 cm from the distal end of the bronchus as far as possible. Three to eight biopsy specimen were obtained in each patient, and the sizes of the specimen were measured.

Eighteen patients underwent TBLC at the MICU bedside through ETT (patients receiving NIPPV support were intubated before the procedure) and supported by pressure control ventilation (FiO_2_ 1.0, PEEP 0 cm H_2_O). Among them, there were nine patients combined with ECMO support during the procedure. The other seven patients underwent TBLC in a hybrid cone-beam computed tomography (CBCT) operating room (OR). The procedure was conducted using rigid bronchoscopy with the patient under general anesthesia and ventilated by a high-frequency jet respirator (FiO_2_ 1.0, respiratory rate 60 bpm, tidal volume 500 mL). In bedside procedures, bronchial balloon blockers (CRE balloon, Boston Scientific Microvasive, Natick, MA, USA) were introduced to the bronchial opening of the target segment. RP-EBUS was used to identify the proper biopsy site during the procedure. A 1.9 mm or 2.4 mm cryoprobe (ERBE, Solingen, Germany) was inserted into the target position. Cryobiopsy was performed (freeze time: 3–6 s) following probe positioning using carbon dioxide as the cryogen. After each biopsy, the bronchial balloon blocker was immediately filled (0.5–1 atm) to stop the bleeding. Two to seven biopsy specimen were obtained in each patient, and the size of each specimen was measured. For patients who underwent TBLC in the hybrid OR, CBCT images (Artis Zee III ceiling, Siemens AG, Munich, Germany) were used to determine the exact position of the cryoprobe before TBLC.

All pathology specimens obtained were examined by experienced pulmonary pathologists. In complex cases, a second pathologist was consulted, and discrepancies were resolved through consensus. The MDD diagnosis was the final clinical diagnosis and was made through discussions among experienced specialists. The MDD team included pulmonary and critical care medicine physicians, radiologists, pathologists, and rheumatologists.

### Outcomes

Complications of procedure were mainly evaluated for bleeding and pneumothorax. Bleeding severity was graded on a scale of 4: no bleeding, mild bleeding (requiring suction for clearance but no other endoscopic procedures), moderate bleeding (requiring endoscopic procedures such as bronchial occlusion-collapse and/or instillation of ice-cold saline), and severe bleeding (causing hemodynamic or respiratory instability, requiring tamponade or other surgical interventions or transfusions) [10]. The diagnosis of pneumothorax was mainly based on the performance of bedside postprocedure X-ray. ΔAPACHE II and ΔSOFA scores are the difference between scores obtained 24 h postprocedure and pre-procedure. Pathological diagnosis was defined as a relatively definitive diagnosis that could be obtained based solely on the biopsy specimen. Multidisciplinary discussion (MDD) diagnosis was defined as the relatively definitive diagnosis that could be obtained after MDD. Treatment modifications and 28-day survival outcomes after the procedure were obtained from the patients’ medical records.

### Statistical analysis

Continuous variables were reported as median within the interquartile range (IQR), considering the small sample size of this study. The Mann–Whitney test was used for continuous variables. Categorical variables were reported as frequencies and percentages. The Fisher exact test or chi-square tests were conducted to compare binary and categorical variables. Cox proportional-hazard regression models were calculated to identify factors associated with poor prognosis. A hazard ratio (HR) was used together with a 95% confidence interval (CI) to measure effect size. For multivariate models, the patients’ baseline conditions, biopsy procedure, safety indicators, and diagnostic information were included for analysis. *p* values < 0.05 were considered statistically significant. The statistical analyses were performed with SPSS statistics software (version 25.0; SPSS Inc., Chicago, IL).

## Results

### Clinical characteristics

In total, 70 acute hypoxemic respiratory failure patients with unknown etiology underwent TBLB or TBLC in a 5-year period review. The median age was 62 (IQR 53–68) years. The percentage of male patients was 57.1% (40/70). Most patients (71.4%, 50/70) had comorbidities based on Charlson Comorbidity Index (CCI). There were 27.1% (19/70) of patients were immunocompromised hosts. About 1/3 (35.7%, 25/70) of patients receive IPPV and/or ECMO support before the biopsy procedure. Biopsy was performed a median of 15 (IQR 10–23) days after the onset of symptoms. The median pre-procedure PFR was 148 (IQR 104–213) mmHg. The median APACHE II and SOFA scores 24 h before the procedure were 14 (IQR 12–22) and 5 (IQR 3–8).

The characteristics of patients underwent TBLB or TBLC are summarized in Table 1. The patients underwent TBLC were more critical, with lower rate of high flow nasal cannula (HFNC) support [36.0%(9/25) vs 68.9%(31/45), *p* = 0.008], higher rate of ECMO support [36.0% (9/25) vs 0, *p* < 0.001], and higher pre-procedure SOFA scores [7.0 (3.0–10.0) vs 4.0 (3.0–7.0), *p* = 0.040] compared with patients underwent TBLB. Pre-procedure platelet count was significantly lower in patients underwent TBLC [159 (97–256) * 10^9^/L vs 249 (167–309) * 10^9^/L, *p* = 0.027].

**Table 1:** Clinical characteristics of patients underwent TBLB or TBLC

	TBLB (n = 45)	TBLC (n = 25)	*p* value
Male (n, %)	28 (62.2)	12 (48.0)	0.249
Age, yrs (Median, IQR)	59 (53.0–68.5)	65 (50.5–68.5)	0.654
CCI (Median, IQR)	1.0 (0.0–2.0)	1.0 (0.5–2.0)	0.643
Immunocompromised host (n, %)	10 (22.2)	9 (36.0)	0.214
*Respiratory Support (n, %)*			
HFNC	31 (68.9)	9 (36.0)	**0.008**
NIPPV	4 (8.9)	1 (4.0)	0.648
IPPV	10 (22.2)	6 (24.0)	0.865
ECMO	0	9 (36.0)	** < 0.001**
PFR, mmHg (Median, IQR)	168 (104–220)	130 (99–178) (n = 20)	0.123
APACHE II score (Median, IQR)	13.0 (11.0–19.5)	20.0 (12.5–24.0)	0.052
SOFA score (Median, IQR)	4.0 (3.0–7.0)	7.0 (3.0–10.0)	**0.040**
PT, s (Median, IQR)	14.5 (13.7–15.3)	14.7 (13.9–16.1)	0.249
APTT, s (Median, IQR)	41.2 (36.5–45.4)	42.0 (35.9–50.1)	0.759
Platelet, *10^9^/L (Median, IQR)	249 (167–309)	159 (97–256)	**0.027**

PFR, APACHE II score, SOFA score, PT, APTT, and platelet count were obtained within 24 h prior to procedure

Bold for statistically significant (*p* value < 0.05)

CCI, Charlson Comorbidity Index; HFNC, high flow nasal cannula; NIPPV, noninvasive positive pressure ventilation; IPPV, invasive positive pressure ventilation; ECMO, extracorporeal membrane oxygenation; PFR, PaO_2_/FiO_2_ ratio; APACHE II score, acute physiology and chronic health evaluation II score; SOFA score, sequential organ failure assessment score; PT, prothrombin time; APTT, partial thromboplastin time

### Safety assessment, diagnosis, and survival

The comparisons about biopsy specimens, safety assessments, diagnostic yields, and survivals between the patients who underwent TBLB and TBLC are shown in Table 2. Crush artifacts and non-alveolar tissue were more common to see in specimens from TBLB in comparison with that from TBLC [crash/atelectasis: 55.6% (25/45) vs 0, *p* < 0.001; non-alveolar tissue: 17.8% (8/45) vs 0, *p* = 0.044]. Traumatic bleedings could be seen in specimens obtained by both TBLB and TBLC, with no significant difference [8.9% (4/45) vs 16.0% (4/25), *p* = 0.443]. None of the patients died because of the procedures. There were no significant differences in overall postprocedure complications between patients who underwent TBLB and TBLC [15.6% (7/45) vs 28.0% (7/25), *p* = 0.212]. But the moderate bleeding event was more common in patients who underwent TBLC [28% (7/25) vs 0, *p* < 0.001], and no patient experienced severe bleeding event after the procedure. However, postprocedure pneumothorax was more frequent in patients who underwent TBLB, although the difference did not reach the statistical difference [13.3% (6/45) vs 0, *p* = 0.056]. One patient developed acute coronary syndrome after TBLB. In addition, the elevation of APACHE II and SOFA scores after procedure were more common in patients who underwent TBLC [rate of ΔAPACHE II score ≥ 5: 16.0% (4/25) vs 0, *p* = 0.006; rate of ΔSOFA score ≥ 3: 16.0% (4/25) vs 2.2% (1/45), *p* = 0.032].

**Table 2:** Comparison of biopsy specimens, safety, diagnosis, and prognosis between the patients underwent TBLB and TBLC

	TBLB (n = 45)	TBLC (n = 25)	*p* value
*Specimen (Median, IQR)*			
Number	4 (2–6)	4 (3–5)	0.380
Length diameter, mm	1.0 (0.5–2.0)	5.3 (4.8–5.6)	** < 0.001**
Short diameter, mm	0.5 (0.5–0.8)	3.7 (3.3–4.3)	** < 0.001**
Artifacts (n, %)			
Crash/atelectasis	25 (55.6)	0	** < 0.001**
Traumatic bleeding	4 (8.9)	4 (16.0)	0.443
Non-alveolar tissue	8 (17.8)	0	**0.044**
Complications (n, %)	7 (15.6)	7 (28.0)	0.212
Moderate bleeding	0	7 (28.0)	** < 0.001**
Severe bleeding	0	0	–
Pneumothorax	6 (13.3)	0	0.056
Others	1 (2.2)	0	1.000
ΔAPACHE II score ≥ 5 (n, %)	0	4 (16.0)	**0.006**
ΔSOFA score ≥ 3 (n, %)	1 (2.2)	4 (16.0)	**0.032**
Pathological diagnosis (n, %)	17 (37.8)	18 (72.0)	**0.006**
MDD diagnosis (n, %)	25 (55.6)	21 (84.0)	**0.016**
Treatment modifications (n, %)	26 (57.8)	21 (84.0)	**0.025**
28-day survival (n, %)	25 (55.6)	16 (64.0)	0.492

APACHE II score, acute physiology and chronic health evaluation II score; SOFA score, sequential organ failure assessment score; MDD, Multidisciplinary discussion

Bold for statistically significant (*p* value < 0.05)

**Fig. 2:**
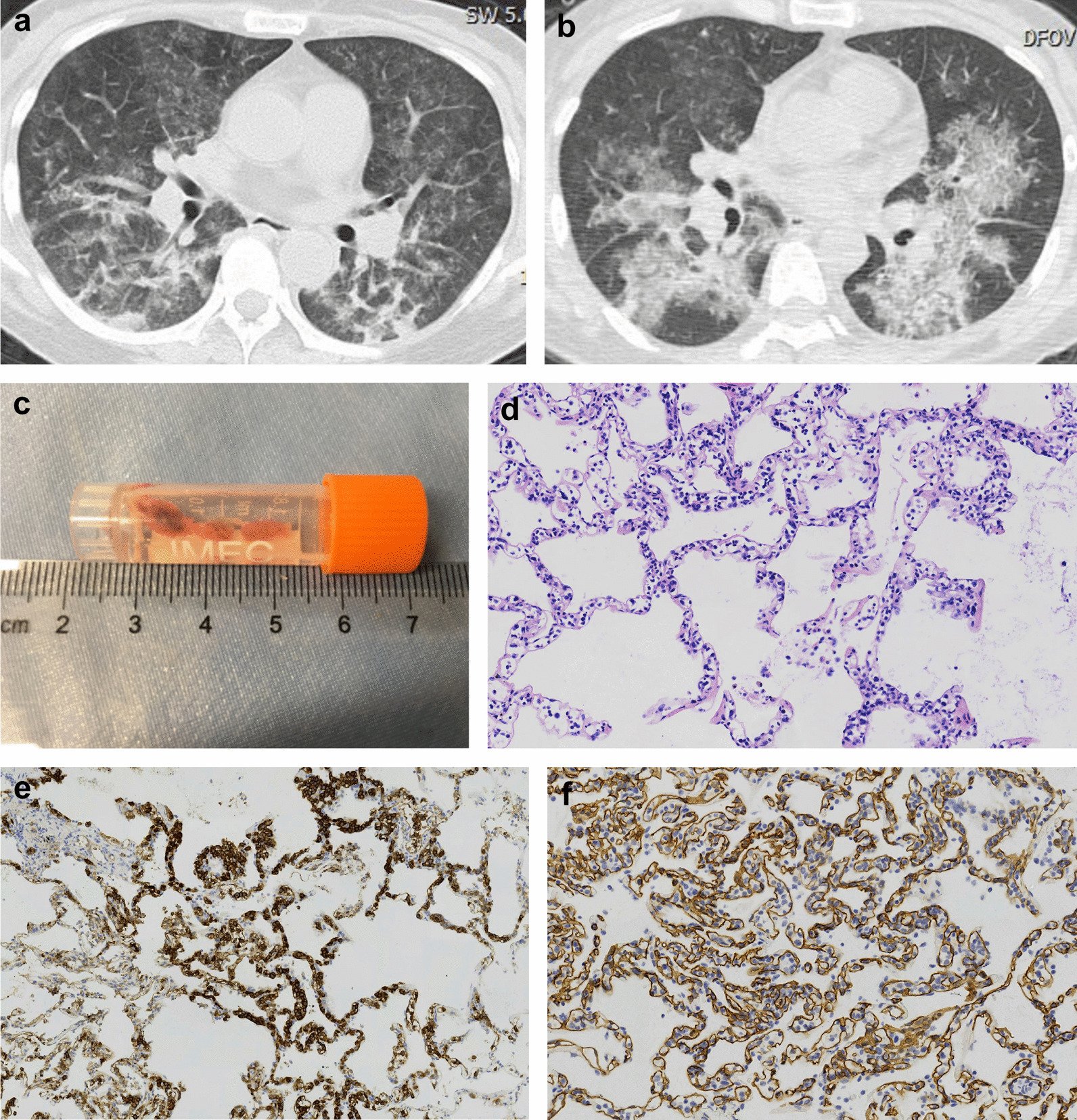
A representational case of patient who underwent TBLC. A 51-year-old female patient was admitted to the MICU with a chief complaint of shortness of breath for two weeks. The patient was diagnosed with immune-mediated necrotizing myopathy three months ago and was treated with oral corticosteroid and mycophenolate mofetil. Chest CT showed bilateral GGOs and consolidations after admission (**a**). Cytomegalovirus nucleic acid was detected in BALF in the initial assessment. However, after treatment of ganciclovir for over one week, the patient's shortness of breath deteriorated. Repeat chest CT still showed progressive pulmonary infiltrations (**b**). Then TBLC was performed under the recommendation in the first MDD (**c**). Pathology of TBLC revealed abundant abnormal lymphocytes infiltrating alveolar septal capillaries and interstitium (**d**). Immunohistochemistry revealed CD20(+) (**e**), CD34 (capillaries+) (**f**), CD3(−), CD79α(+), PAX-5(+). The diagnosis of intravascular large B-cell lymphoma was established according to pathology in the second MDD. Unfortunately, despite receiving life-saving chemotherapy, the patient died 14 days after MICU admission. CT: computed tomography; GGO: ground-glass opacities; BALF: bronchioalveolar lavage fluid; TBLC: transbronchial lung cryobiopsy; MDD: multidisciplinary discussion

Patients who underwent TBLC had a significantly higher rate of pathological diagnosis than those who underwent TBLB [72.0% (18/25) vs 37.8% (17/45), *p* = 0.006]. Detailed diagnosis are listed in Table 3. Organizing pneumonia (9/17) and acute exacerbation of interstitial lung disease (3/17) were the most common pathological diagnosis obtained from TBLB. While organizing pneumonia (7/18) and diffuse alveolar damage (6/18) were the most common pathological diagnosis obtained from TBLC. The rate of MDD diagnosis was also significantly higher in patients who underwent TBLC than in patients who underwent TBLB [84.0% (21/25) vs 55.6% (25/45), *p* = 0.016]. Connective tissue disease associated interstitial lung disease (9/25) and acute exacerbation of interstitial lung disease (6/25) were the most common clinical diagnosis after MDD based on TBLB. While secondary organizing pneumonia (3/21) and acute exacerbation of interstitial lung disease (3/21) were the most common clinical diagnosis after MDD based on TBLC. A representational case of patient who underwent TBLC is shown in Fig. 2. Correspondingly, a higher percentage of patients who underwent TBLC received subsequent treatment modifications compared to patients who underwent TBLB [84.0% (21/25) vs 57.8% (26/45), *p* = 0.025]. The overall 28 day-survival of patients included in the study was 58.6% (41/70). There was no significant difference in 28-day survival between patients who underwent TBLB and TBLC [55.6% (25/45) vs 64.0% (16/25), *p* = 0.492].

**Table 3:** Pathological and multidisciplinary discussion diagnosis in patients underwent TBLB and TBLC

Pathological diagnosis (n = 17)	n (%)
*Patients underwent TBLB*	
Organizing pneumonia	9 (52.9)
Acute exacerbation of interstitial lung disease	3 (17.6)
Pulmonary adenocarcinoma	2 (11.8)
Lymphoma	1 (5.9)
Acute eosinophilic pneumonia	1 (5.9)
Pneumocystis jirovecii pneumonia	1 (5.9)
*Multidisciplinary discussion diagnosis (n* = *25)*	
Connective tissue disease associated interstitial lung disease	9 (36.0)
Acute exacerbation of interstitial lung disease	6 (24.0)
Cryptogenic organizing pneumonia	2 (8.0)
Pulmonary adenocarcinoma	2 (8.0)
Lymphoma	1 (4.0)
Acute eosinophilic pneumonia	1 (4.0)
Acute lung injury	1 (4.0)
Drug induced lung injury	1 (4.0)
Pneumocystis jirovecii pneumonia	1 (4.0)
Adenovirus pneumonia	1 (4.0)

Patients underwent TBLC	n (%)
*Pathological diagnosis (n* = *18)*	
Organizing pneumonia	7 (38.9)
Diffuse alveolar damage	6 (33.3)
Aspiration pneumonia	1 (5.6)
Lymphoma	1 (5.6)
Acute fibrinous organizing pneumonia	1 (5.6)
Acute exacerbation of interstitial lung disease	1 (5.6)
Cytomegalovirus pneumonia	1 (5.6)
*Multidisciplinary discussion diagnosis (n* = *21)*	
Secondary organizing pneumonia	3 (14.3)
Acute exacerbation of interstitial lung disease	3 (14.3)
Connective tissue disease associated interstitial lung disease	2 (9.5)
Aspiration pneumonia	2 (9.5)
Cryptogenic organizing pneumonia	2 (9.5)
Graft versus host disease	2 (9.5)
Acute lung injury	1 (4.8)
Acute fibrinous organizing pneumonia	1 (4.8)
Drug induced lung injury	1 (4.8)
Lymphoma	1 (4.8)
Acute reject reaction after lung transplantation	1 (4.8)
Cytomegalovirus pneumonia	1 (4.8)
Coronavirus pneumonia	1 (4.8)

### Prognostic factors for 28 day-survival

To identify prognostic factors for the patients underwent TBLB or TBLC with unknown etiology of acute hypoxemic respiratory failure, multiple Cox regression analysis was performed. As shown in Table 4, the results of the univariate analysis revealed that pre-procedure APACHE II [HR 1.066 (95% CI 1.013–1.122), *p* = 0.014] and SOFA [HR 1.225 (95% CI 1.094–1.372), *p* < 0.001] scores were associated with poor prognosis. The only prognostic protective factor was treatment modification [HR 0.300 (95% CI 0.144–0.626), *p* = 0.001]. Multivariate regression analysis demonstrated that pre-procedure SOFA score [HR 1.673 (95% CI 1.319–2.122), *p* < 0.001] and postprocedure pneumothorax [HR 7.448 (95% CI 1.649–33.647), *p* = 0.009] were independent risk factors for poor prognosis, while MDD diagnosis [HR 0.193 (95% CI 0.047–0.792), *p* = 0.022] and treatment modification [HR 0.204 (95% CI 0.065–0.638), *p* = 0.006] were prognostic protective factors. Biopsy procedures were not found significant associations with 28-day survival [HR 0.637 (95% CI 0.189–2.151), *p* = 0.468].

**Table 4:** Prognostic factors of 28-day mortality in patients who underwent TBLB or TBLC

	Univariate analysis			Multivariate analysis		
	HR	95% CI	*p* value	HR	95% CI	*p* value
Male	0.741	0.350–1.568	0.433	0.844	0.304–2.344	0.745
Age	1.014	0.986–1.043	0.319	0.995	0.961–1.031	0.797
CCI	1.004	0.840–1.199	0.968	0.929	0.709–1.216	0.591
Immunocompromised host	0.939	0.388–2.128	0.825	1.213	0.396–3.712	0.735
IPPV or ECMO support	1.554	0.747–3.233	0.238	0.700	0.197–2.482	0.581
APACHE II score	1.066	1.013–1.122	**0.014**	0.932	0.845–1.027	0.153
SOFA score	1.225	1.094–1.372	** < 0.001**	1.673	1.319–2.122	** < 0.001**
PT, s	1.125	0.961–1.316	0.161			
APTT, s	1.021	0.981–1.064	0.310			
Platelet, *10^9^/L	0.997	0.994–1.001	0.132			
Procedure	0.749	0.341–1.646	**0.472**	0.637	0.189–2.151	0.468
Number of biopsy	1.067	0.832–1.369	0.610			
Moderate bleeding	0.545	0.139–2.294	**0.408**	0.279	0.035–2.226	0.229
Pneumothorax	2.374	0.824–6.841	**0.109**	7.448	1.649–33.647	**0.009**
ΔAPACHE II ≥ 5	0.594	0.081–4.366	0.608	0.812	0.083–7.935	0.858
ΔSOFA ≥ 3	2.115	0.639–7.006	0.220	5.034	0.992–25.536	0.051
Pathological diagnosis	0.727	0.349–1.511	**0.393**	0.620	0.195–1.967	0.417
MDD diagnosis	0.648	0.309–1.357	**0.250**	0.193	0.047–0.792	**0.022**
Treatment modifications	0.300	0.144–0.626	**0.001**	0.204	0.065–0.638	**0.006**

CCI, Charlson Comorbidity Index; IPPV, invasive positive pressure ventilation; ECMO, Extracorporeal membrane oxygenation; PFR, PaO_2_/FiO_2_ ratio; APACHE II score, acute physiology and chronic health evaluation II score; SOFA score, sequential organ failure assessment score; PT, prothrombin time; APTT, partial thromboplastin time; MDD, Multidisciplinary discussion

Bold for statistically significant (*p* value < 0.05)

## Discussions

Previous studies suggested biopsies such as SLB and TBLB should be considered in patients with undiagnosed acute hypoxemic respiratory failure when initial empirical therapy fails or if empirical therapy is too risky [7]. However, the safety and diagnostic yield of TBLC remains unclear in these patients. To our knowledge, this is the first study comparing the safety and efficacy of TBLB and TBLC in acute hypoxemic respiratory failure with unknown etiology.

In terms of safety assessment, 28% (7/25) of patients who underwent TBLC had moderate bleeding. However, all bleeding events could be effectively controlled by filling bronchial balloon blockers. In previous studies, the rate of complicated moderate to severe bleeding in patients who underwent TBLC was approximate 6.4% [9, 11, 12]. In a small sample study, 70% of patients had mild bleeding [12]. The rate of moderate bleeding complications in our study was higher than that in other studies. The reason for this may be related to the fact that a greater proportion of patients who underwent TBLC in our study received ECMO support, which required adequate anticoagulation. In contrast, none of the patients who underwent TBLC in previous studies received ECMO support. Bleeding events were significantly less in patients who underwent TBLB. Instead, the main complications in these patients were pneumothorax, with a rate of 13.3% (6/45). In previous studies of similar patients, the incidence of pneumothorax after TBLB ranged from 12.5 to 23.7%, which is close to the data of our study [13, 14]. Multivariate Cox regression analysis of our study revealed that pneumothorax is an independent risk factor for poor prognosis in patients with acute hypoxemic respiratory failure. This is one reason why the application of TBLB in mechanically ventilated patients is limited.

The differences between postprocedure and pre-procedure APACHE II and SOFA scores were also compared in our study for evaluating the general impact of procedure in patients with acute hypoxemic respiratory failure. Because of the heterogeneity of the patients included in the different studies, we chose the difference between post and prior procedure APACHE II and SOFA scores to better indicate whether the procedure itself has a prognostic impact on the patients. The results showed that the proportion of those who experienced higher increasing postprocedure APACHE II and SOFA scores was significantly higher in patients who underwent TBLC. A total of four patients in our study had an increasing postprocedure APACHE II score greater than 5, all due to postprocedure intubation duration longer than 24 h. One of them with delayed extubation was related to procedure-induced moderate bleeding. The remaining patients were more related to the severities caused by underlying diseases but not by procedures. A total of four patients in our study had an increasing postprocedure SOFA score greater than 3. Two patients were the ones with increasing postprocedure APACHE II score greater than 5. The other two patients’ postprocedure scores were also not associated with the procedure. Neither univariate nor multivariate Cox regression analyses showed poor prognosis in these patients. More studies are still needed in the future to explore the systemic impact of performing TBLC in critically ill patients.

Our study showed that the pathological diagnostic yield in patients who underwent TBLC (72.0%, 18/25) was significantly higher than patients who underwent TBLB (37.8%, 17/45). In previous studies, the pathological diagnosis rate of TBLC in patients with undiagnosed acute respiratory failure ranged from 88 to 100% [11, 12, 15]. Most studies showed that the pathological diagnosis rate of TBLB in such patients was about 40%, which is similar to the findings of our study [13, 14]. In clinical practice, in addition to the pathological diagnosis, the patients' BALF analysis and serological tests will also provide other diagnostic information. Therefore, multidisciplinary discussions (MDD) are needed to determine the patients' clinical diagnosis, which is widely used in interstitial lung diseases (ILD) diagnosis. A large proportion of patients with acute hypoxemic respiratory failure who have initially excluded infections and other etiologies are diagnosed with ILD. For this reason, we believe that it is necessary to introduce MDD into the diagnostic approach in this group of patients. Our study demonstrated that whether patients received TBLC or TBLB, MDD could improve the diagnostic yield. After MDD, the diagnostic yield of patients who underwent TBLC and TBLB can increase to 84% (21/25) and 55.6% (25/45), respectively. Previous studies have presented that combined BALF analysis can improve the diagnostic value of TBLB and lead to treatment changes in patients with acute hypoxemic respiratory failure [13]. Cox regression analysis of our study also found that the MDD diagnosis and its guided treatment modification were the only protective factors for the prognosis of these patients. There are no studies of surgical lung biopsy studies that have combined MDD to obtain a diagnosis.

Organizing pneumonia (OP) accounted for the highest proportion of pathological diagnoses in our study, both in patients with undiagnosed acute hypoxemic respiratory failure who underwent TBLB and TBLC. Similar results have been discovered in previous studies [5, 14]. Diffuse alveolar damage is another common cause of critical acute respiratory failure patients. In our study, 1/3 of the patients who underwent TBLC were diagnosed with DAD, which is similar to previous studies [11, 12, 15]. While in patients who underwent TBLB, no patients were able to diagnose DAD in our study, similar data were revealed in previous studies [13]. In patients with undiagnosed acute hypoxemic respiratory failure, SLB is considered as the most sensitive procedure in diagnosing DAD, with most studies showing a diagnostic yield of 30–80% [3–7, 16, 17]. The diagnostic yield of DAD by TBLC has approached that of SLB, showing that TBLC is expected to be an appropriate procedure for obtaining a pathological diagnosis in patients with ARDS. Even though all patients in our study were considered to have non-infectious diseases after the initial assessment, a small number of patients were eventually diagnosed with pneumonia, with viral pneumonia accounting for most cases. Similar results have been found in other studies, no matter the method of procedure [3–5, 11, 14, 16].

The multivariate prognostic analysis of our study showed that the main factors affecting the prognosis of undiagnosed patients with acute hypoxemic respiratory failure were pre-procedure status (SOFA score), postprocedure pneumothorax, and whether the diagnosis was established, all of which were closely matched with clinical practice. However, the procedure of biopsies was not found to be significantly associated with the prognosis. TBLC in our study didn’t bring a survival benefit may be due to the group of these patients being more critical prior to the procedure, as reflected by a higher percentage of ECMO support and higher APACHE II and SOFA scores. Rigorous randomized controlled trials are needed to elucidate what type of biopsy is more appropriate for patients with undiagnosed acute hypoxemic respiratory failure in the future.

We acknowledge that our study has some limitations. First, this was a single-center retrospective observational study with relatively small sample size. Second, because the study was not a randomized controlled study, the baselines of patient characteristics were not exactly matched between the two groups. Third, SLB remains the gold standard for definitive diagnosis in acute hypoxemic respiratory failure patients with unknown etiology. However, we failed to make a comparison between SLB and TBLC because of the few experiences of SLB for critically ill patients in our center. Further studies are warranted.

## Conclusions

Compared to TBLB, patients with undiagnosed acute hypoxemic respiratory failure who underwent TBLC had significantly higher rates of pathological diagnosis and MDD diagnosis, which resulted in higher rate of treatment modifications. The overall risk of TBLC was acceptable, even though the risk of moderate bleeding was higher in these patients. It still needs more researches to explore the application of TBLC in critically ill patients with undiagnosed acute hypoxemic respiratory failure in the future.

## Data Availability

The data that support the findings of this study are available from Dr. Guowu Zhou and Dr. Qingyuan Zhan. These data were used under license for the current study, so these data are not publicly available. However, the data are available from the authors upon reasonable request and with permission from corresponding authors.

## Abbreviations

APACHE II score: Acute physiology and chronic health evaluation II score
APTT: Partial thromboplastin time
ARDS: Acute respiratory distress syndrome
BALF: Bronchoalveolar lavage fluid
CBCT: Cone-beam computed tomography
CCI: Charlson Comorbidity Index
CI: Confidence interval
ECMO: Extracorporeal membrane oxygenation
ETT: Endotracheal tube
HFNC: High flow nasal cannula
HR: Hazard ratio
ICU: Intensive care unit
ILD: Interstitial lung disease
IPPV: Invasive positive pressure ventilation
IQR: Interquartile range
MDD: Multidisciplinary discussion diagnosis
MICU: Medical intensive care unit
NIPPV: Noninvasive positive pressure ventilation
OP: Organizing pneumonia
OR: Operating room
PFR: PaO_2_/FiO_2_ ratio
PT: Prothrombin time
RP-EBUS: Radial probe endobronchial ultrasound
SLB: Surgical lung biopsy
SOFA score: Sequential organ failure assessment score
TBLB: Transbronchial lung biopsy
TBLC: Transbronchial lung cryobiopsy
